# Selenium supplementation in patients with peripartum cardiomyopathy: a proof-of-concept trial

**DOI:** 10.1186/s12872-020-01739-z

**Published:** 2020-10-21

**Authors:** Kamilu M. Karaye, Hadiza Sa’idu, Suleiman A. Balarabe, Naser A. Ishaq, Bushra Sanni, Haruna Abubakar, Baba Lawan Mohammed, Tijjani Abdulsalam, Jamilu Tukur, Idris Y. Mohammed

**Affiliations:** 1 Department of Medicine, Bayero University, PO Box 4445, Kano, Nigeria; 2 Department of Medicine, Aminu Kano Teaching Hospital, Kano, Nigeria; 3 Department of Public Health and Clinical Medicine, Umea University, Umeå, Sweden; 4 Department of Medicine, Murtala Mohammed Specialist Hospital, Kano, Nigeria; 5 Department of Medicine, Muhammad Abdullahi Wase Specialist Hospital, Kano, Nigeria; 6 Department of Obstetrics and Gynaecology, Bayero University and Aminu Kano Teaching Hospital, Kano, Nigeria; 7 Department of Chemical Pathology, Bayero University and Aminu Kano Teaching Hospital, Kano, Nigeria

**Keywords:** Peripartum cardiomyopathy, Selenium supplementation, Outcomes, Mortality, PEACE registry, Nigeria

## Abstract

**Background:**

We studied the efficacy and safety of selenium supplementation in patients who had peripartum cardiomyopathy (PPCM) and selenium deficiency.

**Methods:**

We randomly assigned 100 PPCM patients with left ventricular ejection fraction (LVEF) < 45% and selenium deficiency (< 70 μg/L) to receive either oral Selenium (L-selenomethionine) 200 μg/day for 3 months or nothing, in addition to recommended therapy, in an open-label randomised trial. The primary outcome was a composite of persistence of heart failure (HF) symptoms, unrecovered LV systolic function (LVEF < 55%) or death from any cause.

**Results:**

Over a median of 19 months, the primary outcome occurred in 36 of 46 patients (78.3%) in the selenium group and in 43 of 54 patients (79.6%) in the control group (hazard ratio [HR] 0.69; 95% confidence interval [CI] 0.43–1.09; *p* = 0.113). Persistence of HF symptoms occurred in 18 patients (39.1%) in the selenium group and in 37 patients (68.5%) in the control group (HR 0.53; 95% CI 0.30–0.93; *p* = 0.006). LVEF < 55% occurred in 33 patients (71.7%) in the selenium group and in 38 patients (70.4%) in the control group (HR 0.91; 95% CI 0.57–1.45; *p* = 0.944). Death from any cause occurred in 3 patients (6.5%) in the selenium group and in 9 patients (16.7%) in the control group (HR 0.37; 95% CI 0.10–1.37; *p* = 0.137).

**Conclusions:**

In this study, selenium supplementation did not reduce the risk of the primary outcome, but it significantly reduced HF symptoms, and there was a trend towards a reduction of all-cause mortality.

**Clinical trial registration:**

ClinicalTrials.gov Identifier: NCT03081949.

## Background

Peripartum cardiomyopathy (PPCM) is a multifactorial disease with a wide geographical spread, and with significant morbidity and mortality [1–3]. Ventricular reverse remodelling and deaths associated with the disease are more frequent in the first six months after delivery [1–3]. Results from the peripartum cardiomyopathy in Nigeria (PEACE) registry showed significant variation in the burden of PPCM in the country [3]. Incidence rates as high as 1:96 and as low as 1:1350 deliveries were obtained in the Northern and Southern regions of the country respectively [4]. Although reasons for the regional disparity are not yet clear, reports from Niamey (southern Niger Republic) and Kano (northern Nigeria) suggest that selenium deficiency is common in PPCM patients in the regions, with critically low levels of serum selenium in up to 76.9% of the patients [5, 6]. Earlier epidemiological studies in the Keshan and neighbouring counties in northeastern China had established a causal relationship between selenium deficiency and a rapidly progressive and fatal type of cardiomyopathy, the Keshan disease [7]. Subsequently, treatment with sodium selenite showed efficacy in preventing and mitigating the clinical manifestations of the disease [8]. The most commonly used measures of selenium status are plasma and serum selenium concentrations. Quantification of one or more selenoproteins (such as glutathione peroxidase (GPX) and selenoprotein P) is also used as a functional measure of selenium status [9].

To the best of our knowledge, the effects of selenium supplementation on clinical outcomes had not been previously tested in PPCM. We designed this proof-of-concept trial to prospectively study the efficacy and safety of selenium supplementation on clinical outcomes in selenium deficient PPCM patients who had not recovered left ventricular (LV) systolic function at six months after delivery.

## Methods

### Trial design and oversight

The Steering Committee of the trial designed and oversaw the conduct of the trial. The trial was conducted and reported in accordance with the protocol and the statistical analysis plan, which has been published [10]. Patients were recruited from three tertiary-level hospitals in Kano, Nigeria between June 2017 and December 2017: Aminu Kano Teaching Hospital, Murtala Mohammed Specialist Hospital and Mohammed Abdullahi Wase Specialist Hospital. The recruited patients were then followed-up till 31 March 2019. The trial was approved by the Research Ethics Committee at each centre, and the study protocol conforms to the ethical guidelines of the 1975 Declaration of Helsinki as reflected in a priori approval by the hospital's human Research Ethics Committee [11]. The safety of patients in the trial was overseen by an independent Data and Safety Monitoring Committee. The first draft of the manuscript was prepared by the first author, who had unrestricted access to the data, and was reviewed and edited by all the authors. All the authors made the decision to submit the manuscript for publication and testify to the standard of conduct of the trial. The trial was registered in ClinicalTrials.gov (Identifier: NCT03081949).

### Patients

Patients were eligible for inclusion if they had confirmed diagnosis of PPCM as defined by the PPCM working group of the European Society of Cardiology, LV ejection fraction (LVEF) of less than 45% at six months postpartum, serum selenium of less than 70 μg/L, an age of at least 18 years, New York Heart Association (NYHA) functional classes II, III, or IV (for new patients only) and written informed consent [1]. PPCM subjects who were being treated and followed-up at any participating centre before the commencement of the trial were recruited regardless of the presence of symptoms if they had satisfied the other inclusion criteria.

Included patients were requested to receive standard heart failure (HF) drug therapies, including diuretics, beta-blocker, and angiotensin converting enzyme inhibitor (ACEI) or an angiotensin II receptor blocker (ARB) or nitrate-hydralazine combination, unless such use was contraindicated or resulted in unacceptable side effects. In addition, the use of a mineralocorticoid receptor antagonist was encouraged. Additional therapies for HF were prescribed, and drug doses were individually tailored, in accordance with guideline recommendations.

Exclusion criteria included having LVEF ≥ 45% at recruitment, confirmed pregnancy, patients presenting in shock, and those lacking reliable contact phone numbers.

### Trial procedures

After screening for inclusion and exclusion criteria, patients were randomly assigned to receive either selenium (L-selenomethionine) supplement at a dose of 200 μg once daily for 3 months (selenium group) or nothing (control group), in addition to HF standard treatment, using an open-label randomised trial design. The randomisation was carried out by an independent trial monitor who was blinded to the patients’ identities and clinical characteristics, using a random number generator app. Serum selenium and GPX of all the patients were measured at baseline, and the measurement repeated at the end of the trial for the enrolled patients who were alive. Selenium assays were carried out between July and December 2017 at the Central Laboratory of Ahmadu Bello University, Zaria, Nigeria, and in April 2019 at Synlab Reference Laboratory Limited, Lagos, Nigeria, using Inductively Coupled Atomic Emission Spectrometry method. GPX assay was carried out using the Sandwich Enzyme Linked Immunosorbent Assay technique at the Chemical Pathology Laboratory of Aminu Kano Teaching Hospital [10]. Low serum selenium was defined as < 70 μg/L and high serum GPX as > 470 U/L [10]. In this study, hypotension was defined as systolic blood pressure less than 100 mmHg, underweight as body mass index (BMI) less than 18.5 kg/m^2^ and obesity as BMI of more than or equal to 30.0 kg/m^2^, at enrolment.

After randomisation, patients were reevaluated at a monthly interval for three months, and then every three months till 31 March 2019 or until their demise, with a focus on assessment of the trial’s clinical outcomes and side effects of the selenium supplement. Patients’ compliance with the selenium treatment was ensured by using pill counts. Echocardiography and electrocardiography were carried out at recruitment and repeated every six months till the end of the trial. The investigators carrying out these trial procedures were blinded to the treatment allocations of the patient.

### Outcomes

The primary outcome was a composite of persistence of HF symptoms, unrecovered LVEF (less than 55%) or death from any cause. The components of the primary outcome were assessed individually for each group.

For each deceased patient and at each centre, an investigator blinded to the treatment allocation of patients interviewed the first-degree relatives or attending physicians, or the patient’s medical records, to determine the specific cause of death. Deaths were categorised as “unknown” when none of these was successful.

We assessed the rate of all-cause rehospitalisations among the study groups as a secondary outcome.

The pre-specified safety analyses included all adverse events and side effects associated with the discontinuation of the trial treatment.

### Statistical analysis

Proportions, medians with interquartile ranges (IQR) and means with standard deviations were used to summarise subjects’ characteristics, as appropriate. Chi-square, Fisher’s exact test, Student’s *t*- and Mann–Whitney tests were used to compare categorical and continuous variables, as appropriate. Spearman’s correlation coefficient was used to describe the relationship between serum selenium and GPX. The baseline and end of follow-up results for the serum selenium and GPX of the two groups were compared using paired *t* test. Time-to-event data were evaluated with the use of Kaplan–Meier estimates and Cox proportional-hazards models stratified according to the treatment assignment for the opposite outcomes. We used the Cox models to calculate hazard ratios (HR), 95% confidence intervals (CI) and two-sided *p* values, and used a semi-parametric proportional-rates model to calculate total (including recurrent) events. Two-sided *p* value < 0.05 was used as minimum level of statistical significance. The statistical analysis was carried out using Statistical Package for Social Sciences version 23.0 software.

## Results

### Patients

**Fig. 1:**
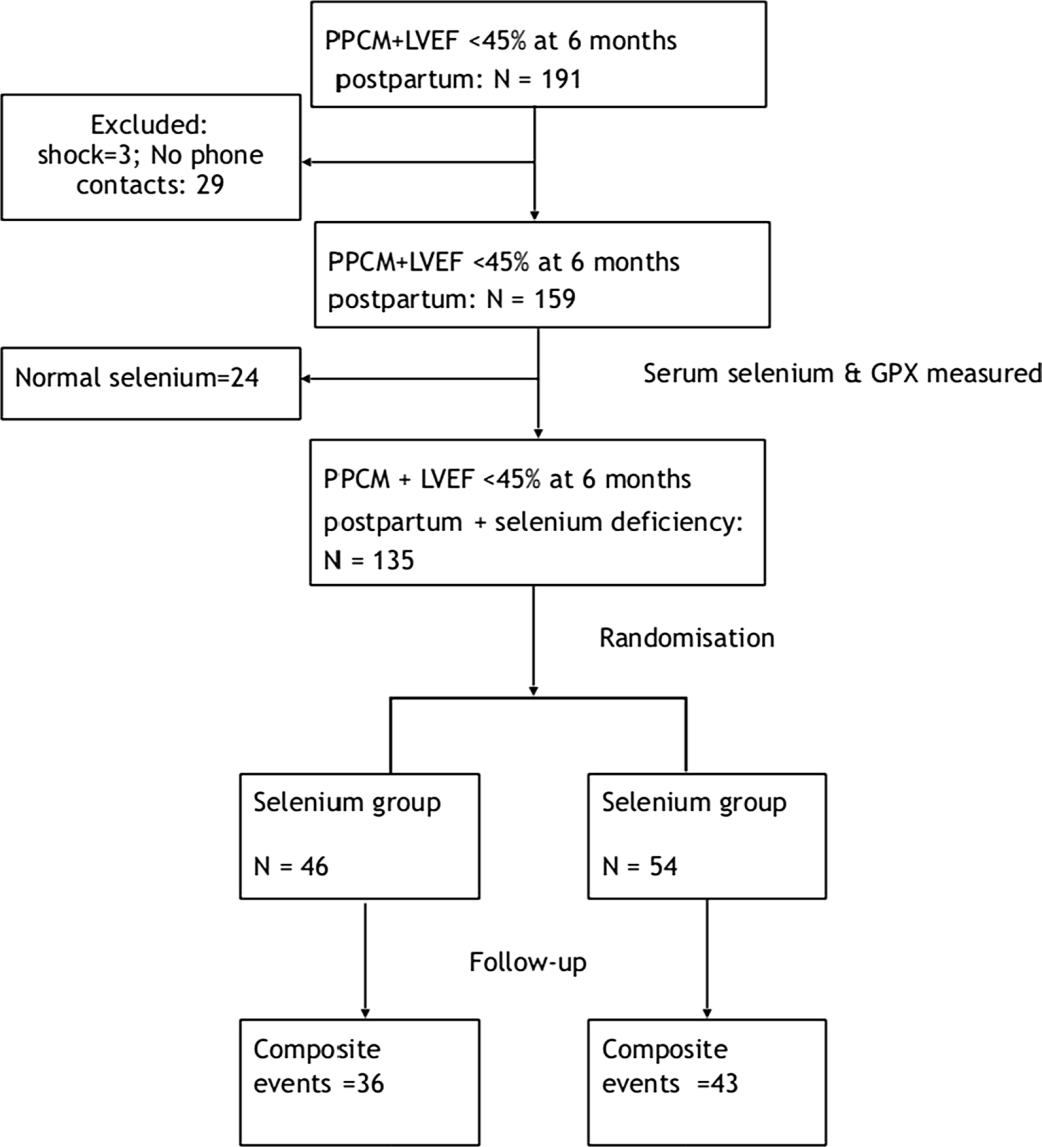
Enrolment and follow-up of patients. *LVEF* left ventricular ejection fraction, *GPX* glutathione peroxidise. All the patients who underwent randomisation were included in the primary analysis

From 12 June 2017 through 1 December 2017, we screened 191 PPCM patients with LVEF < 45% at 6 months postpartum, excluded 53 of them for various reasons and found selenium deficiency (< 70 μg/L) in 135 (84.9%) of them (Fig. 1). Of those with selenium deficiency, we randomly assigned 100 patients to receive either oral selenium 200 μg/day for 3 months (46/100) (selenium group) or nothing (54/100; controls), in addition to recommended HF therapy, in an open-label randomised trial (Fig. 1). All the patients assigned to the selenium group took the supplement for the planned three months, and the median follow up was 19.0 (IQR: 17.0–20.0) months. The characteristics of the patients were well balanced at baseline, and are presented in Table 1. Fifteen (32.6%) patients in the selenium arm and ten (18.5%) in the control arm (*p* = 0.165) were being followed-up at the study sites and were asymptomatic (NYHA I) with LVEF below 45%, at the time of enrolment into the study. All the subjects were accounted for throughout the study without loss to follow-up.

**Table 1:** Baseline characteristics

Characteristic	Selenium supplementation group (n = 46)	Controls (n = 54)	*p* value
Biomarkers			
Serum selenium, μg/L	49.8 ± 10.8	48.1 ± 10.6	0.435
Serum GPX, U/L	326.0 ± 121.9	362.0 ± 144.6	0.323
Serum GPX > 470 U/L	3 (6.5%)	7 (13.0%)	0.466
Demographic and clinical characteristics			
Age, years	29.6 ± 7.3	28.9 ± 8.2	0.664
Age < 20 years	5 (10.9%)	11 (20.4%)	0.277
Age ≥ 35 years	7 (15.2%)	11 (20.4%)	0.536
Hausa/Fulani ethnicity	43 (93.5%)	45 (83.3%)	0.137
Unemployment	32 (69.6%)	46 (85.2%)	0.089
No formal education	14 (30.4%)	25 (46.3%)	0.150
Multiparty	31 (67.4%)	37 (68.5%)	0.904
History of pre-eclampsia	13 (28.3%)	10 (18.5%)	0.249
History of twins	9 (19.6%)	10 (18.5%)	0.894
History of stroke	3 (6.5%)	0	0.094
Body mass index, kg/m^2^	19.8 ± 6.4	20.6 ± 4.8	0.466
Underweight	17 (37.0%)	13 (24.1%)	0.161
Obesity	1 (2.2%)	1 (1.9%)	1.000
NYHA class			0.105
I	15 (32.6%)	10 (18.5%)	
II	19 (41.3%)	26 (48.1%)	
III	7 (15.2%)	12 (22.2%)	
IV	4 (8.7%)	5 (9.3%)	
Heart rate/min	96 ± 17	101 ± 27	0.055
Systolic BP, mmHg	108 ± 17	111 ± 17	0.291
Diastolic BP, mmHg	74 ± 12	77 ± 14	0.203
Hypertension	3 (6.5%)	6 (11.1%)	0.727
Hypotension	19 (41.3%)	20 (37.0%)	0.818
Heart failure treatments			
Loop diuretics	39 (84.8%)	45 (83.3%)	0.804
ACEI or ARB	11 (23.9%)	15 (27.8%)	0.661
Beta-blockers	9 (19.6%)	5 (9.3%)	0.139
Spironolactone	42 (91.3%)	48 (88.9%)	0.750
Digoxin	33 (71.7%)	36 (66.7%)	0.559
Serum creatinine, μmol/L	73.9 ± 34.2	62.7 ± 48.4	0.210
Plasma hemoglobin, g/dl	11.8 ± 3.4	11.3 ± 1.5	0.409
Atrial fibrillation	0	1 (1.9%)	1.000
Echocardiography			
LA dimension, mm	45.1 ± 6.5	45.7 ± 5.8	0.609
LVEDD, mm	62.1 ± 7.2	64.0 ± 7.8	0.212
LVEF, %	30.9 ± 7.1	28.8 ± 8.0	0.186
RV basal diameter, mm	42.7 ± 7.9	42.0 ± 6.9	0.655
TAPSE, mm	14.1 ± 3.7	14.3 ± 2.7	0.756
PASP, mmHg	42.8 ± 19.0	44.0 ± 18.2	0.764
Intracardiac thrombus	2 (4.3%)	2 (3.7%)	0.416

n, number of patients; GPX, glutathione peroxidase; NYHA, New York Heart Association; BP, blood pressure; ACEI, angiotensin converting enzyme inhibitors; ARB, angiotensin II receptor blockers; LA, left atrium; LVEDD, left ventricular end-diastolic dimension; LVEF, LV ejection fraction; RV, right ventricle; TAPSE, tricuspid annular plane systolic excursion; PASP, pulmonary artery systolic pressure; *, *p* value statistically significant. All values were expressed as mean ± standard deviation, or as proportions

Serum selenium was significantly higher at the end of follow-up than at baseline among both the selenium group (49.8 ± 10.8 μg/L vs 85.2 ± 19.0 μg/L respectively, *p* < 0.001) and controls (48.1 ± 10.6 μg/L vs 74.5 ± 17.1 μg/L respectively, *p* < 0.001), representing an increase by 1.71 and 1.55 folds in the serum levels respectively (Table 2). However the corresponding values for GPX (326.0 ± 121.9 U/L vs 341.3 ± 192.7 U/L, *p* = 0.653) among the selenium group and controls (362.0 ± 144.6 U/L vs 307.4 ± 137.4 U/L, *p* = 0.056) did not differ significantly (Table 2).

**Table 2:** Comparison between the baseline and last follow-up characteristics of patients in the selenium and control groups

Characteristic	Selenium supplementation group				Controls			
	Baseline (n = 46)	Final (n = 43)	∆Change	*p* value	Baseline (n = 54)	Final (n = 45)	∆Change	*p* value
Biomarkers								
Serum selenium, μg/L	49.8 ± 10.8	85.2 ± 19.0	+ [35.5]	< 0.001*	48.1 ± 10.6	74.5 ± 17.1	+ [26.4]	< 0.001*
Serum GPX, U/L	326. ± 122	341 ± 193	+ [15]	0.653	362 ± 145	307 ± 137	− 55	0.056
NYHA class								
I	32.6%	58.1%	+ [25.5]%	0.027*	18.5%	17.8%	− 0.7%	0.868
II–IV	67.4%	41.9%	− 25.5%		48.1%	82.2%	+ [34.1]%	
Heart failure treatments								
Loop diuretics	84.8%	23.3%	− 61.5%	< 0.001*	83.3%	40.0%	− 43.3%	< 0.001*
ACEI or ARB	23.9%	58.1%	+ [34.2]%	0.002*	27.8%	51.1%	+ [23.3]%	0.030*
Beta-blockers	19.6%	46.5%	+ [26.9]%	0.013*	9.3%	51.1%	+ [41.8]%	< 0.001*
Spironolactone	91.3%	53.5%	− 37.8%	< 0.001*	88.9%	57.8%	− 31.1%	< 0.001*
Digoxin	71.7%	41.9%	− 29.8%	0.009*	66.7%	55.6%	− 11.1%	0.355
Echocardiography								
LA dimension, mm	45.1 ± 6.5	37.0 ± 5.5	− 8.1	< 0.001*	45.7 ± 5.8	39.0 ± 4.9	− 6.7	< 0.001*
LVEDD, mm	62.1 ± 7.2	55.4 ± 11.7	− 6.7	0.002*	64.0 ± 7.8	55.2 ± 11.2	− 8.8	< 0.001*
LVEF, %	30.9 ± 7.1	45.9 ± 12.2	+ [15.0]	< 0.001*	28.8 ± 8.0	42.0 ± 13.7	+ [13.2]	< 0.001*
RV basal diameter, mm	42.7 ± 7.9	34.0 ± 7.2	− 8.7	< 0.001*	42.0 ± 6.9	34.0 ± 6.4	− 8.0	< 0.001*
TAPSE, mm	14.1 ± 3.7	17.9 ± 4.1	+ [3.8]	< 0.001*	14.3 ± 2.7	16.8 ± 3.1	+ [2.5]	< 0.001*
PASP, mmHg	42.8 ± 19.0	35.1 ± 18.9	− 7.7	0.059	44.0 ± 18.2	47.1 ± 32.4	+ [3.1]	0.551

n, number of patients; GPX, glutathione peroxidase; NYHA, New York Heart Association; ACEI, angiotensin converting enzyme inhibitors; ARB, angiotensin II receptor blockers; LA, left atrium; LVEDD, left ventricular end-diastolic dimension; LVEF, LV ejection fraction; RV, right ventricle; TAPSE, tricuspid annular plane systolic excursion; PASP, pulmonary artery systolic pressure; ∆Change, change in the mean or percentage; *, *p* value statistically significant. All values were expressed as mean ± standard deviation, or as proportions in percentages

At the end of follow-up, serum selenium (85.2 ± 19.0 μg/L vs 74.5 ± 17.1 μg/L, *p* = 0.073) and GPX (341.3 ± 192.7 U/L vs 307.4 ± 137.4 U/L, *p* = 0.533) levels in the selenium and control arms respectively did not differ significantly between the groups.

Treatments received by the patients at baseline are presented in Table 1. At the end of study, prescriptions for patients alive for beta-blockers improved to 46.5% of the selenium group and 51.1% of the controls (*p* = 0.827). Prescriptions for ACEI or ARB also improved during follow-up to 58.1% of the selenium group and 51.1% of the controls (*p* = 0.654). However, sacubitril-valsartan, ivabradine, bromocriptine and intracardiac devices were not prescribed for any of the patients during the study. Patients that received selenium became less symptomatic while the controls became more symptomatic during follow-up (Table 2). Other important differences between the treatments at baseline and at end of follow-up are presented in Table 2.

### Outcomes

**Fig. 2:**
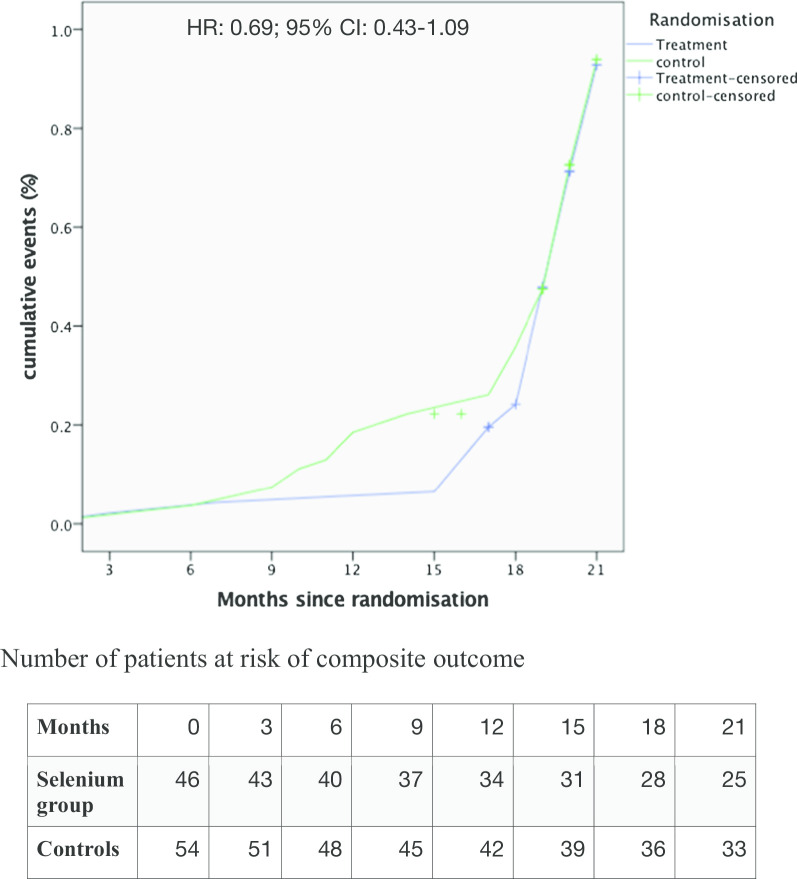
Composite outcome events. Composite outcome: the primary outcome was a composite of persistence of HF symptoms, unrecovered LVEF or death from any cause. The cumulative incidence of the primary outcome was estimated with the use of the Kaplan–Meier method; hazard ratios and 95% confidence interval was estimated with the use of Cox regression models, stratified according to selenium supplementation

The primary outcome (composite of persistence of HF symptoms, unrecovered LV systolic function (LVEF < 55%) or death from any cause) occurred in 36 of 46 patients (78.3%) in the selenium group and in 43 of 54 patients (79.6%) in the control group (HR: 0.69; 95% CI 0.43–1.09; *p* = 0.113) (Fig. 2 and Table 3). HF symptoms persisted in 18 patients (39.1%) in the selenium group and in 37 patients (68.5%) in the control group (HR 0.53; 95% CI 0.30–0.93; *p* = 0.006). LVEF < 55% occurred in 33 patients (71.7%) in the selenium group and in 38 patients (70.4%) in the control group (HR 0.91; 95% CI 0.57–1.45). Death from any cause occurred in three patients (6.5%) in the selenium group and in nine patients (16.7%) in the control group (HR 0.37; 95% CI 0.10–1.37). Of these, six (50.0%; two in selenium group) died of worsening HF, four (33.3%; one in selenium group) died suddenly at home and the remaining two (16.7%) died of an unknown cause. Further analysis showed that the proportion of patients with LVEF below 25% at recruitment was eleven (23.9%) and 22 (40.7%) in the selenium and control groups respectively (0.074).

**Table 3:** Outcomes in selenium and control groups

Outcomes	Selenium supplementation group	Controls	*p* value
Primary outcome			
Composite endpoints	36 (78.3%)	43 (79.6%)	0.867
All-cause deaths	3 (6.5%)	9 (16.7%)	0.137
NYHA II to IV	18 (39.1%)	37 (68.5%)	0.006*
LVEF< 55.0%	33 (71.7%)	38 (70.4%)	0.944
Secondary outcome			
Rehospitalisatons	4 (8.7%)	8 (14.8%)	0.533

NYHA, New York Heart Association; LVEF, LV ejection fraction; *, *p* value statistically significant. All values were expressed as mean ± standard deviation, or as proportions

Given that 32.6% and 18.5% of patients in the selenium and control groups respectively were asymptomatic at enrolment, we subjected the data to further analysis to ascertain the impact of that on the outcomes. The results showed that of the 25 patients that presented with NYHA functional class I, two (8%) died (*p* = 0.378) within the follow-up period and 17 (68%) had LVEF < 55% (*p* = 0.317) at the end of the study, implying a lack of impact of severity of symptoms at enrolment on these outcomes.

For the secondary outcome, four patients (8.7%) in the selenium group and eight (14.8%) (*p* = 0.533) in the control group were rehospitalised during the study.

In the Kaplan–Meier curves for the primary outcome (Fig. 2), the curves for the selenium and control groups began to separate after the 6th month since randomisation and was maximal at 15 months, showing fewer events in the selenium group, but merged again after the 18th month.

### Safety

The selenium supplement was well tolerated, and there were no reported serious adverse effects. However five patients reported that the selenium capsules had an unpleasant smell and taste, which did not lead to the discontinuation of the treatment.

## Discussion

In this randomised, open-label proof-of-consent trial involving PPCM patients with LV systolic dysfunction and selenium deficiency, the risk of the primary composite outcome of persistence of HF symptoms and of LV systolic dysfunction, and death from any cause, was not significantly lowered by selenium supplementation. Of the three components of the composite outcome, selenium supplementation significantly reduced HF symptoms and there was a trend towards a reduction of all-cause mortality. There was no significant difference between the groups for the secondary outcome of all-cause rehospitalisations, although the trend was in favour of selenium supplementation. To the best of our knowledge, this is the first study to test the efficacy of selenium supplementation on clinical outcomes in PPCM patients.

Although selenium supplementation did not reduce the composite outcome at the end of the study, a reduction in favour of selenium supplementation was observed between the 9th and 18th month of follow-up, as shown in the Kaplan Meier curves (Fig. 2). This presumably occurred when the serum selenium levels of patients in the selenium group were significantly higher than in the control group, following the three months supplementation. Unfortunately we could not measure the serum levels of selenium group at the end of the three months treatment because of some prohibitive logistic challenges, to support our hypothesis. Our findings suggest that the three months treatment with selenium was probably too short, and prolonging the treatment until the selenium deficiency is corrected could have significantly lowered the composite outcome at the end of the study.

Studies have shown that in PPCM, adverse outcomes mostly occur in the first six months postpartum, and spontaneous ventricular reverse remodelling does occur even in sub-optimally treated patients [2, 3, 12–14]. In the present study, we randomised PPCM patients who had survived the first six months after delivery, in addition to the other inclusion criteria, to test the effects of selenium supplementation on clinical outcomes, while minimising the impact of the spontaneity of recovery associated with the natural history of the disease. Thus we deliberately recruited the more stable patients to test the efficacy of selenium supplementation on LV functional recovery, though we appreciated that our patient selection could have significantly reduced the magnitude of the efficacy of selenium supplementation on survival and ventricular reverse remodelling. This finding will hopefully be taken into consideration in the design of our future trial on the subject.

Selenium has a wide spectrum of biological effects, and is a critical component of central antioxidant enzymes, including glutathione peroxidases [15]. Thus selenium deficiency could lead to decreased activity of GPX, excessive oxidative stress, myocardial damage and PPCM. Selenium deficiency can also impair many other biologic functions, including those of skeletal muscles, and endocrine and immune systems [15, 16]. Although the effect of selenium on skeletal muscle function was beyond the scope of our study, it is conceivable that it was the direct effect of the selenium supplementation on skeletal muscle function that led to the improvement of symptoms in the present study. We did not find significant correlation between serum selenium and GPX in the present study. Therefore our results support the observation that selenium supplementation may only affect plasma selenium levels and not GPX activity concentration, if study participants were selenium replete before they began taking the selenium supplements [17].

In our study, serum selenium levels increased by 1.71 fold in the selenium group, and by 1.55 fold in the control group, as compared with the baseline values. This was perhaps because at the end of the study, the effect of the selenium supplementation on the serum level had waned, and the general well-being, appetite and feeding of patients in both groups had improved. Serum levels of selenium are determined by many factors. The richest food sources of selenium are seafoods and organ meats [16]. Other sources include muscle meats, cereals and other grains, and dairy products. The concentration of selenium in plants is dependent on the concentration of selenium in the soil and its chemical properties [9]. Consequently, selenium concentrations in plants, as well as in animals that consume these plants, vary significantly by geographic location [9].

The selenium supplement that we used (L-selenomethionine) was well-tolerated without serious adverse effects or the need to withdraw the drug. The choice of the dose of 200 μg once daily of the selenium supplement used in the present study was informed by the findings of a previous study in Eastern United States, in which selenium deficient subjects took and tolerated the same dose well for a mean of eight years [18]. We are therefore confident that prolonging the duration of the treatment will also be well tolerated by patients.

Majority of patients in the present study were referred to the study centres while on treatment with loop diuretics, spironolactone and digoxin, but only 19.6% and 23.9% of the selenium group and 9.3% and 27.8% of the controls were on beta-blockers and ACEI or ARB respectively. The reasons for these poor prescriptions of guideline-directed HF therapies will include the fact that the referring hospitals and clinics lacked the equipment for echocardiography and the expertise to diagnose and start management of PPCM itself. However, prescriptions for these drugs improved significantly during follow-up. Beta-blockers, ACEI, ARB and other recommended HF therapies have been shown to reduce mortality and morbidity in symptomatic patients with HF and reduced ejection fraction, and are strongly recommended for treatment of PPCM patients except when contraindicated [19]. Bromocriptine could be beneficial to some PPCM patients in the acute phase of the disease and may be considered [20]. Implantable cardioverter defibrillator as well as cardiac resynchronisation therapy are recommended for PPCM patients presenting with severe LV dysfunction for more than six months despite optimal medical therapy [19]. Wearable and subcutaneous implantable cardioverter defibrillators represent an alternative to transvenous systems in PPCM patients because they can be more easily removed/extracted if cardiac function recovers.

## Conclusion

In this randomised, open-label proof-of-concept trial involving PPCM patients with LV systolic dysfunction and selenium deficiency, the risk of the primary composite outcome of persistence of HF symptoms and of LV systolic dysfunction, and death from any cause, was not significantly lowered by selenium supplementation. However, selenium supplementation significantly reduced HF symptoms and there was a trend towards a reduction of all-cause mortality. There was no significant difference between the groups for the secondary outcome of all-cause rehospitalisations, although the trend was in favour of selenium supplementation.

## Limitations

This proof-of-concept trial has limitations that are inherent to its study design. We used an open-label study design that has potential for bias, and did not repeat measurement of the serum selenium levels at the end of treatment phase with selenium, but only at the end of the study. We also did not include a placebo-arm, partly because of the study’s proof-of-concept trial design, and partly because of limited funding. B-type natriuretic peptide measurement could have also been useful in the prognostication of the patients but it was not readily available at the study centres. We hope to overcome these limitations that were partly caused by our modest funding in our future studies on the subject matter.

## Data Availability

The datasets used and/or analysed during the current study are available from the corresponding author (KM Karaye, kkaraye@yahoo.co.uk) on reasonable request. We do not have access to data repositories open to the public at the moment.

## Abbreviations

ACEI: Angiotensin converting enzyme inhibitor
ARB: Angiotensin II receptor blocker
BMI: Body mass index
CI: Confidence interval
GPX: Glutathione peroxidase
HF: Heart failure
HR: Hazard ratio
IQR: Interquartile range
LV: Left ventricle
LV: Left ventricular ejection fraction
NYHA: New York heart failure association
PEACE: Peripartum cardiomyopathy in Nigeria
PPCM: Peripartum cardiomyopathy
