# Correction to: Selenium supplementation in patients with peripartum cardiomyopathy: a proof‑of‑concept trial

**DOI:** 10.1186/s12872-020-01782-w

**Published:** 2021-01-04

**Authors:** Kamilu M. Karaye, Hadiza Sa’idu, Suleiman A. Balarabe, Naser A. Ishaq, Bushra Sanni, Haruna Abubakar, Baba Lawan Mohammed, Tijjani Abdulsalam, Jamilu Tukur, Idris Y. Mohammed

**Affiliations:** 1 Department of Medicine, Bayero University, PO Box 4445, Kano, Nigeria; 2 Department of Medicine, Aminu Kano Teaching Hospital, Kano, Nigeria; 3 Department of Public Health and Clinical Medicine, Umea University, Umeå, Sweden; 4 Department of Medicine, Murtala Mohammed Specialist Hospital, Kano, Nigeria; 5 Department of Medicine, Muhammad Abdullahi Wase Specialist Hospital, Kano, Nigeria; 6 Department of Obstetrics and Gynaecology, Bayero University and Aminu Kano Teaching Hospital, Kano, Nigeria; 7 Department of Chemical Pathology, Bayero University and Aminu Kano Teaching Hospital, Kano, Nigeria

## Correction to: BMC Cardiovasc Disord (2020) 20:457, 10.1186/s12872-020-01739-z

Following publication of the original article [1], the author identified two errors in the article body.

1. "selenium group" should be replaced with "controls" in Fig. 1.
2. "consent" should be replaced with "concept" in the first sentence of the Discussion section.

The original article [1] has been corrected.

## References

[1] Karaye (2020). Selenium supplementation in patients with peripartum cardiomyopathy: a proof‑of‑concept trial. BMC Cardiovasc Disord.

